# Supplementary material for: Modeling glioblastoma heterogeneity as a dynamic network of cell states
Source: Mol Syst Biol. 2021 Sep 16;17(9):e10105. doi: 10.15252/msb.202010105 (PMC8444284; doi:10.15252/msb.202010105)
Supplement: Supplementary file 6 — Source Data for Figure 5 [file MSB-17-e10105-s004.zip › Figure5A_sourcedata/GSEA_3017/nosigdb_stateA.GseaPreranked.1622795026485/OLSEN_MESENCHYMAL.html]

Details for gene set OLSEN\_MESENCHYMAL[GSEA]

|  || Dataset | state53017 |
| Phenotype | NoPhenotypeAvailable |
| Upregulated in class | na\_neg |
| GeneSet | OLSEN\_MESENCHYMAL |
| Enrichment Score (ES) | -0.65520483 |
| Normalized Enrichment Score (NES) | -2.829903 |
| Nominal p-value | 0.0 |
| FDR q-value | 0.0 |
| FWER p-Value | 0.0 |
Table: GSEA Results Summary

  

Fig 1: Enrichment plot: OLSEN\_MESENCHYMAL      
 Profile of the Running ES Score & Positions of GeneSet Members on the Rank Ordered List

  

| PROBE | GENE SYMBOL | GENE\_TITLE | RANK IN GENE LIST | RANK METRIC SCORE | RUNNING ES | CORE ENRICHMENT || 1 | VIM |  |  | 46 | 0.543 | -0.0162 | No |
| 2 | RPL41 |  |  | 436 | 0.285 | -0.4040 | No |
| 3 | MT2A |  |  | 473 | 0.275 | -0.4254 | No |
| 4 | ZFP36L1 |  |  | 532 | 0.264 | -0.4703 | No |
| 5 | CD99 |  |  | 604 | -0.252 | -0.5295 | No |
| 6 | THY1 |  |  | 663 | -0.277 | -0.5736 | No |
| 7 | S100A6 |  |  | 717 | -0.313 | -0.6105 | No |
| 8 | EMP1 |  |  | 724 | -0.322 | -0.5980 | No |
| 9 | IGFBP7 |  |  | 780 | -0.368 | -0.6338 | Yes |
| 10 | SPARC |  |  | 782 | -0.369 | -0.6134 | Yes |
| 11 | LHFPL6 |  |  | 796 | -0.381 | -0.6047 | Yes |
| 12 | NPC2 |  |  | 800 | -0.383 | -0.5855 | Yes |
| 13 | FTL |  |  | 825 | -0.409 | -0.5867 | Yes |
| 14 | PRRX1 |  |  | 829 | -0.413 | -0.5658 | Yes |
| 15 | TMSB4X |  |  | 850 | -0.443 | -0.5608 | Yes |
| 16 | IFITM2 |  |  | 864 | -0.460 | -0.5476 | Yes |
| 17 | PRSS23 |  |  | 874 | -0.486 | -0.5286 | Yes |
| 18 | CALD1 |  |  | 889 | -0.527 | -0.5125 | Yes |
| 19 | CST3 |  |  | 933 | -0.680 | -0.5176 | Yes |
| 20 | IFITM3 |  |  | 954 | -0.830 | -0.4901 | Yes |
| 21 | COL5A2 |  |  | 958 | -0.856 | -0.4434 | Yes |
| 22 | ANXA1 |  |  | 972 | -1.018 | -0.3977 | Yes |
| 23 | ID3 |  |  | 981 | -1.505 | -0.3184 | Yes |
| 24 | IGFBP5 |  |  | 983 | -1.595 | -0.2266 | Yes |
| 25 | CCL2 |  |  | 985 | -1.881 | -0.1182 | Yes |
| 26 | NEAT1 |  |  | 986 | -2.049 | 0.0010 | Yes |
Table: GSEA details [plain text format]

  

Fig 2: OLSEN\_MESENCHYMAL: Random ES distribution      
 Gene set null distribution of ES for **OLSEN\_MESENCHYMAL**
